# CRISPR/Cas9 Edition of the *F9* Gene in Human Mesenchymal Stem Cells for Hemophilia B Therapy

**DOI:** 10.3390/life14121640

**Published:** 2024-12-11

**Authors:** Irving Jair Lara-Navarro, Luis Felipe Jave-Suárez, Juan Antonio Marchal, Ana Rebeca Jaloma-Cruz

**Affiliations:** 1 Doctorado en Genética Humana, Centro Universitario de Ciencias de la Salud, Universidad de Guadalajara, Guadalajara 44340, Jalisco, Mexico; irvinglaran@gmail.com; 2 División de Inmunología, Centro de Investigación Biomédica de Occidente, Instituto Mexicano del Seguro Social, Guadalajara 44340, Jalisco, Mexico; luis.jave@academicos.udg.mx; 3 Departamento de Anatomía y Embriología Humana, Universidad de Granada, 18012 Granada, Spain; jmarchal@ugr.es; 4 Instituto de Investigación Biosanitaria de Granada (ibs. GRANADA), 18100 Granada, Spain; 5 Excellence Research Unit Modelling Nature (MNat), BioFab i3D-Biofabricación y 3D (Bio) Printing Laboratory Granada, Universidad de Granada, 18100 Granada, Spain; 6 División de Genética, Centro de Investigación Biomédica de Occidente, Instituto Mexicano del Seguro Social, Guadalajara 44340, Jalisco, Mexico

**Keywords:** hemophilia B, lentiviral vector, CRISPR/Cas9 system, mesenchymal stem cells, gene therapy

## Abstract

Hemophilia B is a genetic disorder characterized by clotting factor IX deficiency and bleeding in joints and muscles. Current treatments involve intravenous infusion of plasma-derived products or recombinant proteins, which have limited efficacy due to the short half-life of infused proteins. Recently, gene therapy for bleeding disorders has offered a potential solution. This study aimed to develop an in vitro gene therapy model using the CRISPR/Cas9 system to incorporate the *F9* cDNA in human mesenchymal stem cells (hMSCs) to produce clotting factor IX. RNA guide sequences targeting the promoter-exon 1 region of the *F9* gene were designed to incorporate a wild-type *F9* cDNA into the cells. Knockin was performed with the CRISPR/Cas9 system and pDONOR-CMV/cDNAF9/IRES/EGFP vector template recombination in Lenti-X HEK293 cells and MSCs. A lentiviral *F9* cDNA vector was designed as a FIX secretor model to validate the CRISPR/Cas9 system. Results showed successful gene editing and *F9* expression in both cell models, although editing efficiency was lower in hMSCs. Future investigations will focus on improving gene editing efficiency using different transfection conditions or hybrid methodologies. This study demonstrates the potential of CRISPR/Cas9-based gene therapy in hMSCs as a target for hemophilia B. Further optimizations are required to translate these findings into clinical applications.

## 1. Introduction

Hemophilia B (HB) is an X-linked recessive disorder caused by pathogenic variants in the *F9* gene that result in functional deficiency of coagulation factor IX (FIX). Although HB has a worldwide prevalence of 1 in 30,000 male live births [1], in Mexico only 732 patients with this disease have been reported [2]. The primary clinical manifestation is prolonged bleeding episodes that primarily affect joints and muscles and are due to a low or absent FIX activity resulting in weak clots [3]. To date, there are 1094 *F9* pathogenic variants registered in the Factor IX Variant Database [4].

Currently, intravenous administration of recombinant or plasma-derived factor IX is the gold standard treatment for acute episodes and as prophylaxis in severe cases to prevent prolonged bleeding and joint damage.

In the last decade, a new generation of recombinant proteins with structural modifications to gain functional activity or increase their half-life has innovated the treatment for hemophilia [5]. In hemophilia A and, to a lesser degree, in HB, the current non-invasive and non-factor therapy based on antibodies with a long-lasting effect has demonstrated great efficiency. However, despite their improved safety and potential for providing a better quality of life, recombinant proteins are still partially effective because of their limited half-life [6].

Correction of pathogenic variants promises to be the definitive cure for HB [7]. Indeed, initial gene therapy trials using Adeno-associated vectors (AAV) have increased the plasma FIX levels by 2–5% and reduced bleeding episodes [5,8]. Moreover, the efficacy of this approach has been improved by the introduction of high-activity FIX variants (such as Padua, FIXR388L) that achieve higher FIX plasmatic levels (4%) over longer periods (at least 52 weeks) [9]. However, limitations of AAV include a reduced DNA packing capacity (up to 5 kb), poor genomic integration, possible immune reactions, and restricted application to individuals who develop neutralizing antibodies after exposure to environmental AAVs with serotypes like those of the most used vectors, namely AAV2/8 and AAV5.

The Clustered Regularly Interspaced Short Palindromic Repeats (CRISPR) system and its associated proteins (Cas) use RNA guides and endonucleases to recognize and cleave specific genome sequences [10]. A previous work has shown the promising potential of human mesenchymal stem cells (hMSCs) as target cells for gene therapy of HB via nucleofection [11]. Based on the efficiency of CRISPR/Cas9 gene editing to correct pathogenic variants, it is crucial to test and validate a model for HB.

The CRISPR/Cas9 system has recently been proposed as a promising, low-toxicity strategy for genome editing of MSCs [12]. These cells offer a great advantage as a therapeutic target due to their proliferative potential into different tissues and because they provide multiple non-invasive sites for producing FIX useful for treating HB in a novel way. Otherwise, this approach has potential in vivo application, as shown by the successful results of gene editing via CRISPR/Cas 9 with nucleofection of an *F9*-specific mutation in mouse embryonic stem cells but also present in an HB patient [13].

In this regard, we propose that hMSCs obtained from HB patients’ adipose tissue may be a minimally invasive and convenient strategy to probe in vitro the potential of the CRISPR/Cas9 system to produce a functional FIX. Hopefully, autologous transplantation of the corrected cells will complement or even replace current treatments.

## 2. Materials and Methods

### 2.1. Cell Lines Selection and Culture Conditions

Experimental assays were performed with two commercial human cell lines. Lenti-X HEK293 (Takara Bio Inc.©, Kusatsu, Japan) is a genetically modified human embryonic kidney cell line, which has been widely employed as a gold standard for the production and packing of viral particles. StemPro^®^ Human Adipose-Derived Stem Cells, Catalog Numbers R7788110 and R7788115 (Invitrogen™, Waltham, MD, USA), were selected as a feasible source of hMSCs intended to be the therapeutic target due to their proliferative and differentiation capacity, expansion potential, adherence, secretion, and immunoregulation properties [11].

We used the culture media recommended by the manufacturers to preserve the morphological traits and functional properties of each cell line. For Lenti-X HEK293, we used the Dulbecco’s Modified Eagle Medium (DMEM) with high glucose (4.5 g/L) (Cat. number 11965084, Thermofisher Scientific™, Waltham, MA, USA) supplemented with bovine serum albumin (BSA) (5%) and antibiotics (penicillin/streptomycin, 10%). For the hMSCs, we used the specific culture medium MesenPRO RS™ (Cat. Number 12746012, Thermofisher Scientific™) composed of a basal medium, growth supplement (prepared at the time of culture), and antibiotics. As guaranteed by the manufacturer, this medium preserves the undifferentiated properties of cells throughout multiple passages. For reproducibility of the results, all experiments were performed at the fourth passage of both cell lines.

### 2.2. RNA Guides Design

Breaking-Cas (CNB-CSIC, Madrid, Spain) and CRISPOR software v. 4.0(UCSC, Santa Cruz, CA, USA [14] were employed to design guide RNA sequences for flanking and editing the promoter-exon 1 site of the *F9* gene using the CRISPR/Cas9 system. Forward and reverse RNA guide sequences were GCTCTCTGACAAAGATACGG (TGG) and GTACTTACCAACCTGCGTGC (TGG) for 5′ and 3′ strands, respectively; they were ordered to IDT™, Integrated DNA Technologies, Inc., Coralville, IA, USA.

### 2.3. Vector Design and Production

The design of the lentiviral particle vector and the homologous recombination template was performed on the platform of VectorBuilder Inc.© software version 2.1.940 (Chicago, IL 60609, USA), according to its recommendations. Both vectors contained the *F9* cDNA to produce human FIX and the EGFP protein sequence as a positivity reporter. Then, the homologous recombination template and lentiviral vector with cDNA of the *F9* gene were obtained (the vector IDs are VB201022-1213kyv and VB200607-1366uru, which can be used to retrieve detailed information about the vector on vectorbuilder.com) accessed on 11 October 2020.

### 2.4. Genome Cell Knockout

Genome knockout in Lenti-X HEK293 and hMSCs was performed in vitro using ribonucleoprotein (RNP) complex formed by Cas9 nickase (Cas9n) enzyme (IDT™, Integrated DNA Technologies, Inc., IA, USA) and guide RNAs (IDT™, Integrated DNA Technologies, Inc., IA, USA) for the *F9* gene. In vitro digestion was observed by acrylamide electrophoresis. The target sequence used for digestion was 795 base pairs in length. After digestion with the RNP complex, three bands were expected (795, 501, and 294 bp), indicating that the forward and reverse RNA guides successfully direct the cleavage at the interested target sites (promoter-exon 1 of *F9* gene).

### 2.5. CRISPR/Cas9 Knockin Assay

Transfections of the RNP complex and recombination template on Lenti-X HEK293 and hHMSCs were achieved using Lipofectamine™ CRISPRMAX™ Cas9 (wild type) Transfection Reagent (Invitrogen™, MD, USA) for gene editing once cell confluence was greater than 70%. For experimental conditions, we followed the manufacturer’s protocol. After 48 h, knockin models were analyzed by fluorescence microscopy for EGFP protein expression. We carried out an initial knockin experiment by triplicate for both Lenti-X HEK-293 and hMSCs.

### 2.6. Lentivirus Particles Productio

We used the Lipofectamine™ 3000 protocol (Invitrogen™, MD, USA) to obtain lentiviral particles from Lenti-X HEK293 cells transfected with the pLV-Puro-CMV-hF9 vector. After 48 h of transfection, culture medium was collected and a qualitative test was performed (Lenti-X™ GoStix™, Takara Bio Inc.©, Kusatsu, Japan).

### 2.7. Overexpression FIX Models

Departing from 100,000 cells/well, Lenti-X HEK293 and hMSCs were cultured for two days to reach 80% of confluence and were incubated with 1.25 × 10^5^ and 2.5 × 10^5^ IFU lentiviral particles, respectively, for 72 h to obtain FIX-producing models. The number of lentiviral particles was established according to a minimal 90% viability of each cell line after transduction. After lentiviral exposition, the cells were treated for another 72 h with 1 μg/mL of puromycin (GIBCO, Life Technologies™ Corporation, Carlsbad, CA, USA) for cloning selection.

### 2.8. Validation of Lentiviral Secretory FIX Models

Quantitative analysis of *F9* mRNA expression in secretory models infected with lentiviral particles (Lenti-X HEK293 and hMSCs) was performed using real-time PCR in LightCycle^r©^ 96 Instrument (Hoffmann-La Roche Ltd.^©^, Basel, BS, Switzerland). *GAPDH* gene expression was used as an internal control, and relative expression was calculated using the ΔΔCt method. Additionally, FIX protein quantification was performed in the cell culture medium of each model. Supernatant samples were collected and analyzed from zero up to 72 h post-infection with viral particles using an ELISA assay (VisuLize™ Factor IX Antigen, Affinity Biologicals™ Inc., Ancaster, ON, Canada).

## 3. Results

### 3.1. Knockout and Knockin Cellular Models with CRISPR/Cas9 System

**Figure 1:**
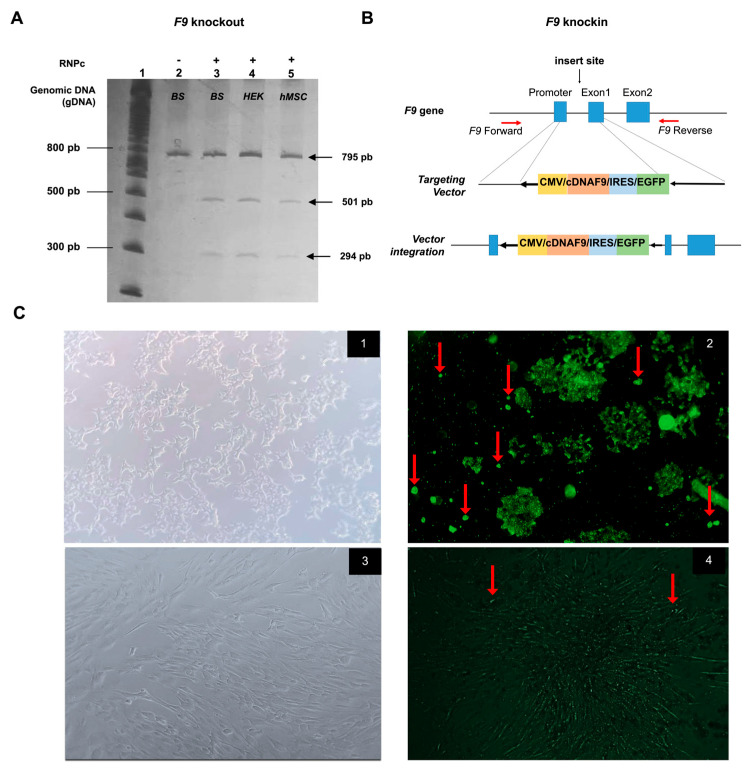
*F9* Knockout and knockin in HEK293 and human MSCs. (**A**) Representative gel of RNP complex cleavage assay for *F9* knockout in genomic DNA (gDNA) showing three bands after 37 °C incubation: (1) 100 bp Ladder; (2) gDNA from blood sample (gDNA_BS), negative control, without RNP complex (-RNPc) (3) gDNA_BS +RNPc; (4) gDNA_Lenti-X HEK293 (HEK) +RNPc; (5) gDNA_hMSCs +RNPc. (**B**) Gene pattern diagram of CMV/cDNAF9/IRES/EGFP template insertion for knockin in the promoter-exon 1 of *F9* gene. (**C**) Images of cell models before (1,3) and after (2,4) RNP complex transfection. EGFP positive clones are indicated with red arrows. The experiments were performed using the fourth passage of HEK293 and hMSCs cell lines.

Knockout in vitro assay demonstrated that RNA guides act on the *F9* promoter-exon 1 site in Lenti-X HEK293 and hMSCs cells genomic DNA (Figure 1A,B). Acrylamide gel electrophoresis revealed that genomic DNA was not cleaved when RNA guides were not added. Conversely, the addition of the RNP complex resulted in the pattern of three bands expected whenever the forward and reverse RNA guides cleaved in both directions. For knockin, twenty-four hours after transfection with the RNP complex and DNA template, cells that incorporated the CMV/cDNAF9/IRES/EGFP template (Figure 1C) tested positive for EGFP in Lenti-X HEK293 and human mesenchymal cells (photos 2 and 4, respectively). To assess the knockin efficiency, we directly counted EGFP-positive cells per visual field at 400× magnification in a confluent cell culture. We found 5–10 EGFP-positive cells/100 HEK-293 total cells (5% of knockin efficiency), and 1–2 EGFP-positive cells/100 hMSCs total cells (1% of knockin efficiency) (Figure 1C, photos 2 and 4, respectively).

### 3.2. Human F9 Gene and FIX Protein Secretion Models with Lentiviral Vectors

**Figure 2:**
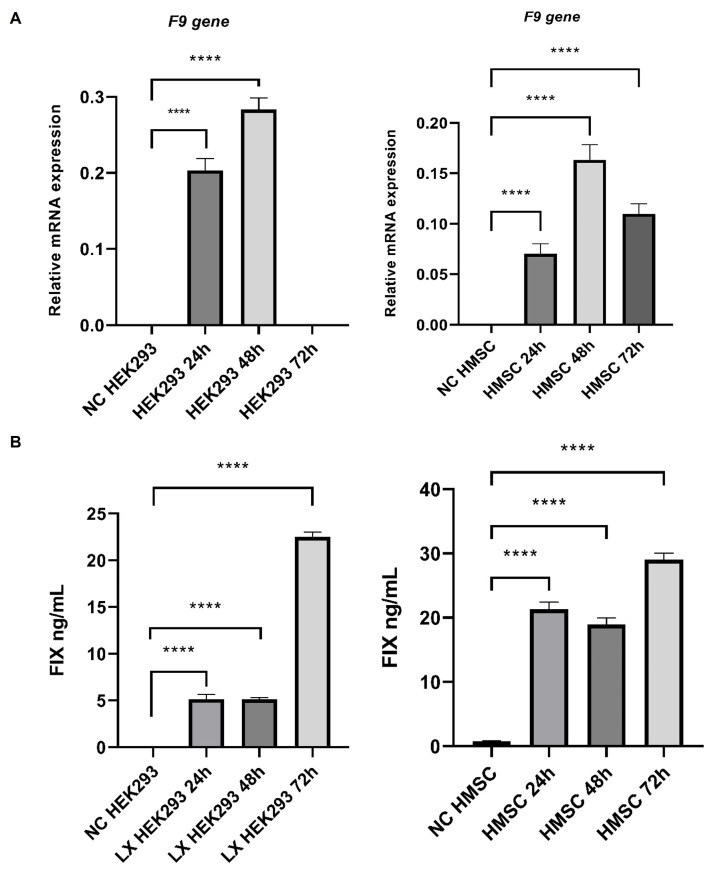
Overexpression of *F9* gene and human FIX protein in Lenti-X HEK293 (LXHEK293) and human mesenchymal cells (HMSC). (**A**) Relative mRNA levels of *F9* gene (normalized to *GAPDH*) measured at 24, 48 and 72 h (n = 3 for each group). (**B**) Human FIX protein in the supernatant of cellular models measured at 24, 48, and 72 h (n = 3 for each group). Error bar means SD of biological triplicate samples. Pairwise comparisons confirmed significant differences in NC models and infected cells (LX HEK293, *p* < 0.0001; HMSC, *p* < 0.0001), data are shown as **** *p* < 0.0001. NC: Negative control.

Real-time PCR assays of *F9* gene expression in Lenti-X HEK293 and human mesenchymal cells models revealed elevated amounts of mRNA after 24 h of culture post-infection (Figure 2A). Likewise, ELISA assays confirmed the secretion of FIX in both models (Figure 2B).

## 4. Discussion

In this study, we used vectors carrying the therapeutic gene and viral particles to generate overexpression models of human FIX protein as a reference. Additionally, gene editing with the CRISPR/Cas9 system and guide sequences of the *F9* gene was performed in two cell lines. Unlike viral systems, CRISPR/Cas9 editing yields stable and long-lasting expression of factor IX and hence represents a promising therapy for HB as has already been demonstrated in mammalian experimental models [15] and, using MSC cells as the therapeutic target, in mouse models [13] and human MSCs [12].

Novel strategies, such as ex vivo editing of hematopoietic stem cells with lentivirus and AAV, have been explored for hemophilia B treatment in different clinical trials [16]. Our results in Lenti-X HEK293 and human MSCs showed that lentiviral vectors promote the expression of the human IX protein for 72 h (Figure 1) with levels like those reported previously [17].

Our results were also consistent with the production of human FIX protein by MSCs transfected with lentiviruses [18]. However, Dodd et al. achieved higher production levels for 45 days (6000 ng/mL) by including an intron 1 fragment of the *F9* gene in their lentiviral vector, which improved FIX production [19]. Although our short-time model yielded a proportional production (29 ng/mL), the long-term production must be evaluated to validate our strategy and optimize the FIX expression. These results indicated that Lenti-X HEK293 and human MSCs are excellent models for recombinant protein production, especially the MSCs due to their immunoregulatory, differentiation capability, and proliferative proprieties [11].

Human MSCs were validated as a therapeutic target for CRISPR/Cas9 editing comparable to the gold standard Lenti-X HEK293 model. MSCs, due to their high protein production capacity and ease of transfection, are ideal for correcting pathogenic variants [13] and for producing therapeutic proteins in diseases such as HB [20,21]. The CRISPR/Cas9 strategy with homology-directed repair templates allows precise integration of transgenes at specific sites [22], making this model a promising tool for the development of new gene therapies. Despite achieving knockout and knockin events in Lenti-X HEK293 and human MSCs by means of the CRISPR/Cas9 system, a low expression of EGFP indicated a low editing efficiency under our experimental conditions. Although our initial attempts to express the EGFP protein have yielded lower than expected results, this study confirms the ability of CRISPR gene editing in MSCs to produce the FIX protein potentially suitable for clinical application in HB [14].

According to other reports, a low editing efficiency in MSCs can be overcome with a Cas9 RNP-mediated system in combination with neon transfection and nucleofection, an approach that confirms the usefulness of hybrid strategies to increase both gene expression and cell viability, in contrast to plasmid transfection alone [12], as we used in our protocol.

Recent studies [23] demonstrate that strategies such as the use of AAV for donor templates and liposome-mediated introduction of RNP complexes into the cells can optimize gene and protein expression. Therefore, we plan to convert our approach into a hybrid strategy combining liposomes for the RNP complex and AAV to introduce the donor template as a more effective way to increase editing efficiency. The use of liposomes to introduce RNP complexes and adenoviral vectors to introduce the *F9* donor template has restored stable FIX blood levels in hemophilia B mice [8]. However, it is crucial to consider the potential risks of viral vectors, such as off-target gene editing and exacerbation of the immune response [24].

CRISPR/Cas9 offers an excellent potential for treating monogenic diseases such as hemophilia B. The combination of this technology with MSCs and their validation with viral models opens new doors for gene therapies. However, despite global advancements in clinical trials, Mexico does not have a specific normative institution or gene therapy regulation. The lack of this framework hinders the development and clinical application of these therapies in our country [25].

## 5. Conclusions

Bioinformatic software for RNAs and vector synthesis allows their obtaining and production. Human FIX secretor Lenti-X HEK293 and MSCs models produced with lentiviral particles are a reliable reference for *F9* gene expression and FIX protein production as well as to compare and validate the CRISPR/Cas9 system. Even though knockout and knockin outcomes were achieved with the CRISPR system in both cell models assessed here, it is necessary to improve the knockin by modifying the transfection conditions of RNP or using hybrid methodologies, which will be addressed in future investigations.

## Data Availability

All data generated or analyzed during this study are included in this article. The raw data supporting the conclusions of this article will be made available by the authors on request.
